# Impact of chronic obstructive pulmonary disease (COPD) in the Asia-Pacific region: the EPIC Asia population-based survey

**DOI:** 10.1186/s12930-015-0020-9

**Published:** 2015-04-23

**Authors:** Sam Lim, David Chi-Leung Lam, Abdul Razak Muttalif, Faisal Yunus, Somkiat Wongtim, Le Thi Tuyet Lan, Vikram Shetty, Romeo Chu, Jinping Zheng, Diahn-Warng Perng, Teresita de Guia

**Affiliations:** Duke-NUS Graduate School of Medicine, Singapore, Singapore; Department of Medicine, University of Hong Kong, Pokfulam , Hong Kong; Institute of Respiratory Medicine, Kuala Lumpur, Malaysia; Department of Pulmonology and Respiratory Medicine, Universitas Indonesia (FMUI), Jakarta, Indonesia; Department of Internal Medicine, Chulalongkorn University, Bangkok, Thailand; Respiratory Care Center, University Medical Center, Ho Chi Minh City, Vietnam; Takeda Pharmaceuticals (Asia-Pacific) Pte. Ltd, Singapore, Singapore; State Key Lab of Respiratory Disease, National Clinical Research Center for Respiratory Disease, Guangzhou Institute of Respiratory Disease, First Affiliated Hospital of Guangzhou Medical University, Guangzhou, People’s Republic of China; Chest Department, Taipei Veterans General Hospital and School of Medicine, National Yang Ming University, Taipei, Taiwan; Division of Pulmonary and Critical Care Medicine, Philippine Heart Center, Quezon City, Philippines

**Keywords:** Chronic obstructive pulmonary disease (COPD), Asia-Pacific, Population-based, Survey, Prevalence, Exacerbations, Impact, Quality of life (QoL)

## Abstract

**Background:**

Chronic obstructive pulmonary disease (COPD) is a clinical syndrome encompassing a group of chronic, progressive, and debilitating respiratory conditions, that are characterized by incompletely reversible airflow limitation. Within the Asia-Pacific region, prevalence estimates have been derived using various protocols and study methods, and there is little data on the impact of COPD exacerbations. This study aimed to provide a comprehensive picture of the current prevalence and burden of COPD in this region.

**Methods:**

A population-based survey was conducted in nine Asia-Pacific territories between 01 February 2012 and 16 May 2012. Overall, 112,330 households were screened to identify eligible subjects (aged ≥40 years, with a physician diagnosis of COPD, chronic bronchitis or emphysema, or with identifiable symptoms of chronic bronchitis). Out of a sample of 69,279 individuals aged ≥40 years, 4,289 subjects with COPD were identified. Data were collected via face-to-face interviews or by fixed-line telephone, using a structured questionnaire. A total of 1,841 completed questionnaires were analyzed.

**Results:**

The overall estimated COPD prevalence was 6.2%, with 19.1% of subjects having severe COPD. In the 12 months prior to the survey, nearly half of all subjects (46%) had experienced exacerbations, and 19% had been hospitalized as a result of their condition. When subjects were asked about the impact of their condition on employment, 23% said their condition kept them from working, and 42% felt that their condition limited their ability to work or their activities. Of those who reported taking prescription drugs, 20% did not know the name of the drugs they were taking. Prescription of oral corticosteroids was common, with 44% of subjects having used these during the previous year to manage their respiratory symptoms; in contrast, inhaler use was low (25%). Only 37% of subjects had taken a lung function test, and the majority (89%) of those tested did not know their test results.

**Conclusions:**

Across the Asia-Pacific territories surveyed, the prevalence of COPD is high, indicating a substantial socioeconomic burden. Our findings suggest that there is considerable room for improvement in the management of COPD, and highlight a need to enhance patient and physician education in the region.

**Electronic supplementary material:**

The online version of this article (doi:10.1186/s12930-015-0020-9) contains supplementary material, which is available to authorized users.

## Background

Chronic obstructive pulmonary disease (COPD) is a clinical syndrome that encompasses a group of chronic, progressive, and debilitating respiratory conditions, including emphysema and chronic bronchitis. COPD is the fourth leading cause of global mortality [1], and its prevalence is predicted to rise [2,3]. Despite the wealth of information regarding its causes, pathophysiology, and treatment options, the disease has historically been under-diagnosed and under-reported, especially within the Asia-Pacific region [1,4].

COPD is characterized by persistent, progressive airflow limitation, and is often accompanied by cough and increased sputum production [4]. Airflow limitation is associated with chronic inflammation in the lungs and is principally caused by long-term exposure to airborne irritants such as cigarette smoke. In the Asia-Pacific region, smoke from biomass fuels and industrial toxins are also known to be problematic risk factors [5-7]. The symptoms of COPD cause significant impairment of quality of life (QoL), including breathlessness, anxiety, and physical limitations, resulting in days of missed work [8].

COPD exacerbations, consisting of an acute worsening of the usual symptoms beyond normal day-to-day variation, can be particularly debilitating [4]. While some exacerbations may be relatively mild and go unreported [9], in severe cases they can be particularly debilitating, requiring weeks for full recovery [10]. Recent studies have indicated that there may be exacerbation-specific phenotypes [11,12], and that cough and sputum (chronic bronchitis) are associated with a greater exacerbation frequency [13,14].

Previous studies of the prevalence of COPD and its exacerbations in the Asia-Pacific have focused on individual countries or cities [15-19], or relied on mathematical modeling [20]. The Epidemiology and Impact of COPD (EPIC) Asia survey is the first population-based COPD survey to cover nine Asia-Pacific regions using the same study design and questionnaire. We collected data on COPD exacerbation and its indicators, such as cough and sputum, as these aspects of the disease have not been well documented in this region. We also considered measures of disease reporting, disease severity, and socioeconomic factors, along with treatment and management practices. The aim was to gain further insight regarding the current prevalence and burden of COPD in the Asia-Pacific region.

## Methods

**Figure 1:**
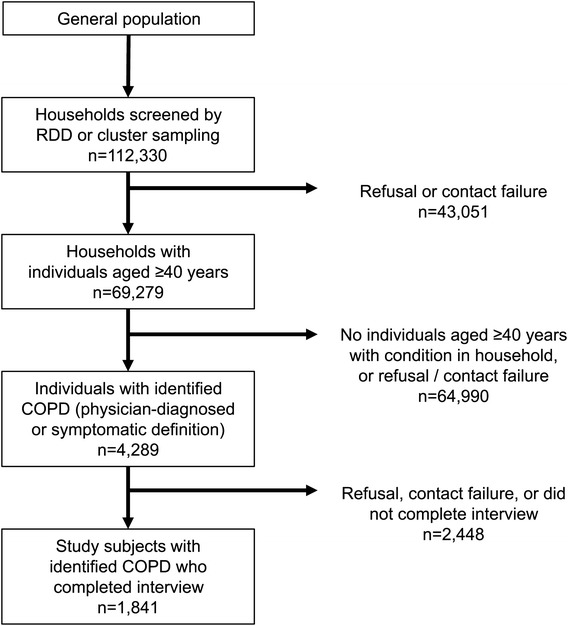
Sampling strategy and response rate for the EPIC Asia survey. ‘Study subjects’ refers to the subset of individuals aged ≥40 years who were identified as having COPD, based on the definitions used in this study (see Methods), and who completed the questionnaire.

The EPIC survey was conducted between 01 February 2012 and 16 May 2012 in nine Asia-Pacific territories: China, Hong Kong, and Taiwan (North Asia), and Indonesia, Malaysia, the Philippines, Singapore, Thailand, and Vietnam (Southeast Asia). Household screening and subject selection were carried out by telephone or face-to-face interviews (Figure 1; Additional file 1: Table S1). A structured questionnaire was then administered to eligible subjects.

Households were screened by random sampling to reduce selection bias. Fixed-line random digit dialing (RDD) sampling was conducted in regions with a high coverage of fixed telephone lines. Numbers were chosen based on randomly selected blocks of numbers. In the remaining regions, area probability (cluster) sampling was conducted face-to-face (FF) in subjects’ homes. Areas were divided into primary sampling units, which were then randomly selected, and a block or building was chosen as a starting point.

Individuals who met the following criteria were eligible for inclusion in the survey: individuals aged ≥40 years, who reported either a physician diagnosis of the following: emphysema, chronic bronchitis, COPD, chronic obstructive airways disease, or chronic obstructive lung disease; or who met the following symptomatic definition of chronic bronchitis: production of phlegm or mucus from the lungs on all or most days for three consecutive months. As this was a community survey which did not include any research intervention, no ethical approval was required. Verbal consent was obtained from all subjects prior to participation in the survey. Participation was voluntary. Subjects were informed that their results may be published in scientific articles and their responses would be kept both anonymous and confidential.

The structured questionnaire used for data collection was based on those used in previous studies [21,22], with additional questions to capture information on exacerbations. This questionnaire was developed and implemented by Abt SRBI, Inc., on behalf of Takeda Pharmaceuticals. The same questionnaire and study design were utilized across all territories. Where necessary, the English language questionnaire was translated by a local translator to the local language and then reviewed by an independent translator with health research experience, as well as by local medical experts. During data collection, potential bias was mitigated by random sampling within households containing more than one eligible individual, multiple contact attempts to reduce contact failure, and quality control during interviews. The fieldwork teams received extensive training in all aspects of administering the questionnaire. Mock and pre-test interviews were used to confirm training standards and identify areas for modification.

Only eligible subjects who completed the study questionnaire were included in the analyses, which involved standard descriptive statistics. Disease prevalence was calculated as (100 × number of eligible subjects ÷ number of individuals aged ≥40 years), and expressed as a percentage. Severe COPD was defined based on subjects’ recall of COPD classification by their physicians, according to the Global Initiative for Chronic Obstructive Lung Disease (GOLD) criteria (GOLD grade III or IV), or using the following symptomatic definition: presence of the symptoms of chronic bronchitis, together with two or more exacerbations in the previous 12 months.

## Results

### Subject demographics

In the nine territories surveyed, a total of 112,330 households were screened, identifying 69,279 households with one or more individuals aged ≥40 years. Of the 69,279 individuals aged ≥40 years, 4,289 either had a physician’s diagnosis of COPD or met the symptomatic definition used in this survey. Of these 4,289 subjects with identified COPD, a total of 1,841 subjects completed the questionnaire (Figure 1). Almost half of the study population (44%) was between 45 and 60 years of age, and 56% were female. For country-specific figures, please refer to Additional file 1: Table S1. The mean interview duration was 41 minutes.

### Estimated prevalence of COPD and related conditions

Based on the above criteria, the overall estimated prevalence of COPD was 6.2%, ranging from 4.5% in Indonesia to 9.5% in Taiwan (Table 1). The proportion of subjects with a physician diagnosis of COPD was 59%, with the remainder having the symptomatic definition. Physician diagnosis was higher in North Asia (72–93%) than in Southeast Asia (19–60%), with the exception of Vietnam (92%). Overall, 19.1% of the subjects met the definition of the severe phenotype used in this study, ranging from 12.5% in Malaysia to 37.5% in Vietnam (Table 1). The majority of subjects reported their COPD classification as GOLD stage I or II (34.1% and 37.9%, respectively), with only a minority reporting it as stage III (9.3%) or IV (2.1%) (Table 1). However, Southeast Asian countries had higher percentages of subjects with severe or very severe disease. The mean MRC dyspnea score [23] was 2.3.

**Table 1:** **Subject demographics**

	**Overall**	**North Asia**			**Southeast Asia**					
**Self-reported characteristic**	**EPIC Asia n = 1,841**	**China n = 215**	**Hong Kong n = 205**	**Taiwan n = 207**	**Indonesia n = 200**	**Malaysia n = 200**	**Philippines n = 200**	**Singapore n = 200**	**Thailand n = 214**	**Vietnam n = 200**
**Age (years), %**										
40–44	26	45	50	46	18	12	16	18	18	10
45–49	19	28	28	30	14	14	15	10	14	18
50–54	14	9	10	8	18	19	13	9	15	22
55–59	11	4	4	3	13	16	14	13	15	15
60–64	11	6	2	4	16	15	19	18	11	12
65+	20	7	5	9	22	26	23	34	28	25
**Gender, %**										
Female	56	40	47	60	49	67	63	59	64	60
**Work status, %**										
Employed	47	66	75	68	36	31	37	30	40	40
**Smoking, %**										
Never smoked on a regular basis	58	45	53	60	53	69	56	68	55	64
**COPD prevalence, %**										
EPIC Asia	6.2	8.1	7.7	9.5	4.5	5.1	4.2	5.9	5.3	9.4
Prevalence estimation models [20]	6.3	6.5	3.5	5.4	5.6	4.7	3.5	6.3	5.0	6.7
Severe symptomatic phenotype	19.1	13.0	16.1	24.2	20.5	12.5	13.0	20.0	15.9	37.5
Diagnosed vs symptomatic (Mean age of diagnosis/yrs)	59 vs 41 (44 vs 40)	72 vs 28 (43 vs 41)	90 vs 10 (42 vs 42)	93 vs 7 (43 vs 46)	60 vs 40 (42 vs 35)	33 vs 67 (44 vs 40)	40 vs 60 (46 vs 38)	19 vs 81 (46 vs 38)	33 vs 67 (45 vs 41)	92 vs 8 (50 vs 50)
MRC dyspnea score (mean) [23]	2.3	1.8	2.2	2.2	2.6	2.4	2.6	2.4	2.1	2.5
**GOLD stage of severity** [4]**, %**										
Stage I – Mild	34.1	44.7	52.2	44.0	28.0	26.5	30.5	27.0	20.6	32.5
Stage II – Moderate	37.9	32.1	32.2	34.8	44.0	34.0	35.5	48.5	36.0	44.5
Stage III – Severe	9.3	5.6	2.9	4.8	16.5	9.5	5.0	13.5	9.3	17.5
Stage IV – Very severe	2.1	0.9	1.0	0.5	<0.5	2.5	4.0	4.0	4.2	2.0
Not diagnosed/not told	11.2	14.4	10.2	13.5	6.5	14.5	6.5	4.5	28.0	1.5
Don’t know	5.3	2.3	1.0	2.4	5.0	13.0	18.5	2.5	1.9	2.0
**Other frequent health conditions, %**										
None	45	61	61	63	47	47	29	39	18	40
Nasal allergies	13	18	20	15	2	3	11	2	32	14
Arthritis	11	7	11	6	5	6	19	4	13	24
Asthma	19	1	5	2	17	21	40	33	48	7
Diabetes	10	5	4	4	8	18	10	24	17	4
Hypertension	21	9	8	5	13	23	23	36	42	28
Heart disease	5	<1	2	1	7	11	6	5	8	5
**General health status, %**										
Excellent	2	2	<1	1	9	<1	<1	<1	<1	<1
Very good	3	9	2	5	2	6	3	2	<1	<1
Good	17	17	14	12	13	25	20	19	26	6
Fair	48	61	70	69	33	22	58	33	53	37
Poor	23	9	13	10	35	40	19	15	19	54
Very poor	6	2	1	4	8	7	<1	32	2	5

Subjects from each of the nine territories were sampled either by telephone, using random digit dialing (RDD), or face-to-face (FF) interviews in their local language, to identify individuals who had either received a physician diagnosis of COPD or met the symptomatic criteria used (see Methods).

A total of 1,841 subjects completed the study questionnaire for the EPIC Asia survey. All figures are percentages of subjects from the respective territory, with the exception of mean age of COPD diagnosis and mean MRC dyspnea score.

### Health status and disease symptoms

There was a clear North/Southeast division in reported health status (Table 1). The proportion of subjects who report that their health was ‘poor’ or worse ranged from 11–14% in North Asia to 19–59% in Southeast Asia. Similarly, the proportion who considered their general health to be ‘fair’ varied from 22% in Malaysia to 70% in Hong Kong.

Overall, a substantial proportion of subjects (33–53%) reported symptoms typical of COPD at least twice a week during their worst month in the previous 12 months (Additional file 2: Figure S1). Overall, 34% of the subjects reported that physical exertion instigated their COPD symptoms (Additional file 2: Figure S1D).

### Exacerbations and unplanned healthcare utilization

**Figure 2:**
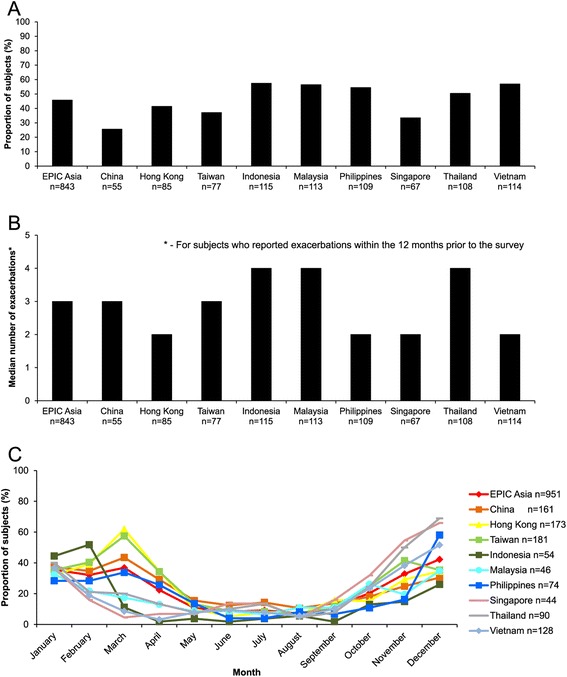
Prevalence, frequency, and seasonal variation of exacerbations. **(A)** Proportion of study subjects who reported experiencing one or more exacerbations within the 12 months prior to the survey. **(B)** Median number of exacerbations reported by subjects over this period. **(C)** Proportion of subjects who reported exacerbations within each month over this period.

Almost half (46%) of all subjects reported experiencing exacerbations in the 12 months prior to the survey (Figure 2A). The frequency and seasonal variation of reported exacerbations is shown in Figure 2B and C. The median number of exacerbations reported was 3, with all territories falling within the range of 2–4 (Figure 2B). Exacerbations occurred more frequently between October and January (Figure 2C). For the North Asian territories and the Philippines, an increase in exacerbations was also seen in the months from February to April. Subjects reported worsening of their disease symptoms during exacerbations, with over half of the subjects reporting coughing up phlegm or sputum, or coughing during the day (Additional file 2: Figure S2).

**Figure 3:**
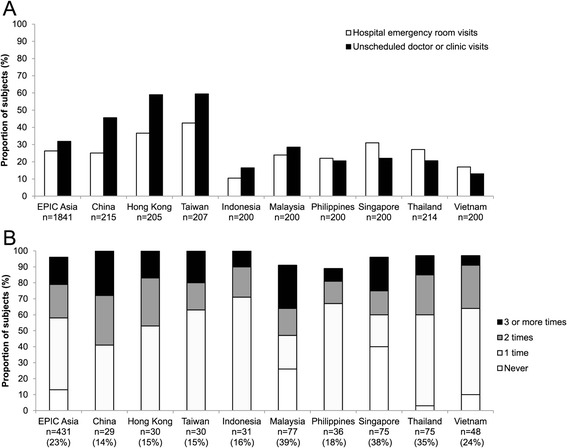
Unplanned healthcare utilization. **(A)** Proportion of subjects who either visited a hospital emergency room or made unscheduled visit(s) to a doctor or clinic as a result of their condition, in the 12 months prior to the survey. **(B)** Subjects who had ever been hospitalized because of their condition (n; %) were asked how many times they had been hospitalized in the previous 12 months. The proportions of subjects who had been hospitalized 0, 1, 2, or 3 or more times are indicated by the shading within each bar. Results are shown only for subjects who were able to report the number of times they had been hospitalized.

Overall, a sizable proportion of study subjects reported visiting a hospital emergency room (26%), or making other unscheduled visits to a doctor or clinic (32%) in the previous 12 months, as a result of their condition (Figure 3A). China (46%), Hong Kong (59%), and Taiwan (59%) recorded the highest percentages of unscheduled doctor or clinic visits (Figure 3A). Overall, 19% of study subjects reported being hospitalized in the previous 12 months as a result of their condition (Figure 3B).

### Impact of disease on employment and daily activities

**Figure 4:**
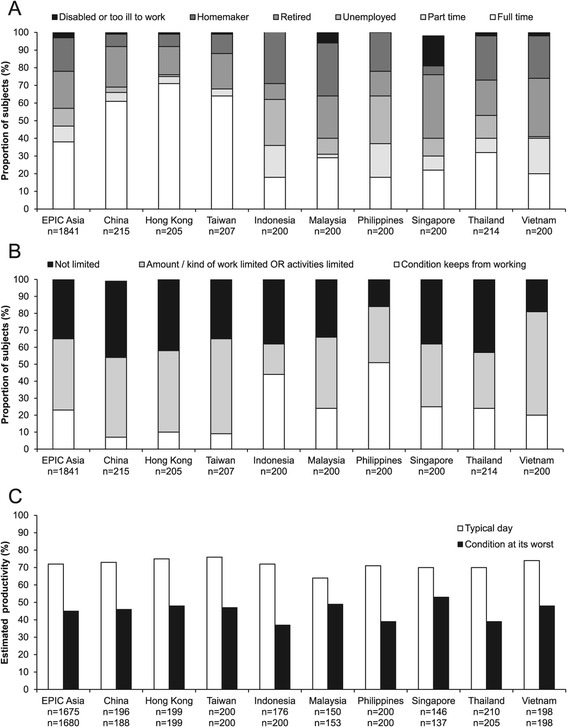
Impact of disease on employment and work productivity. **(A)** Employment status of study subjects. **(B)** Proportion of subjects who reported that their condition kept them from working, limited the kind or amount of work they could do, or limited their activities. **(C)** Subjects’ estimated level of productivity on a typical day, and on a day when symptoms are at their worst. Upper row of n values: subjects who answered the question regarding productivity on a typical day; lower row of n values: subjects who answered the question regarding their worst day.

Less than half (47%) of all subjects were employed (either full- or part-time), with the employment rate in North Asia being higher (66–75%) than in Southeast Asia (30–40%) (Figure 4A). A substantial proportion of subjects (23%) reported that their condition kept them from working (Figure 4B), particularly in Indonesia (44%) and in the Philippines (51%). In addition, 42% felt that their condition limited the kind or amount of work they could do, or limited their activities (Figure 4B). Subjects were also asked to estimate their levels of productivity on a typical day, and on a day when symptoms were at their worst. Overall, average estimated productivity was 72% on a typical day, falling to 45% when the condition was at its worst (Figure 4C).

The impact of disease symptoms on daily activities was also explored (Additional file 2: Figure S3). Notably, 39% reported that their condition limited normal physical activities, such as walking. A substantial proportion of subjects (27–49%) said that their respiratory symptoms placed restrictions on a range of daily activities, including sleep, household chores, social or recreational activities, or affected their sex life (Additional file 2: Figure S3, A–C).

### Disease management

**Figure 5:**
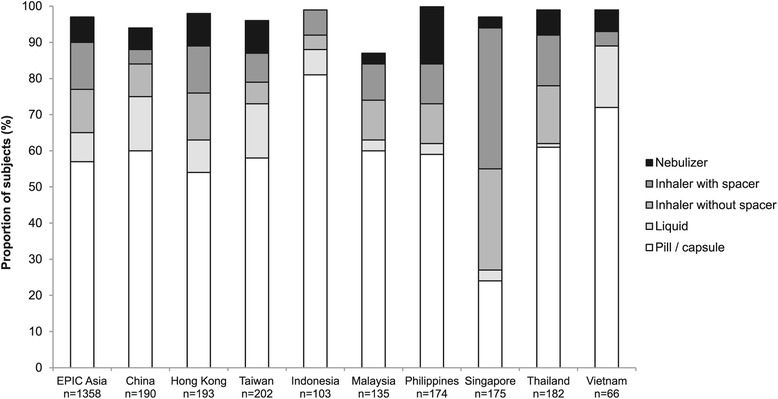
Delivery format of prescribed medication. Subjects who reported taking a prescription drug were asked about the delivery format of their medication. Numbers below the bars indicate the total number of valid answers for this question, for the corresponding territory. Results are shown only for subjects who were able to report the delivery format of their medication.

**Figure 6:**
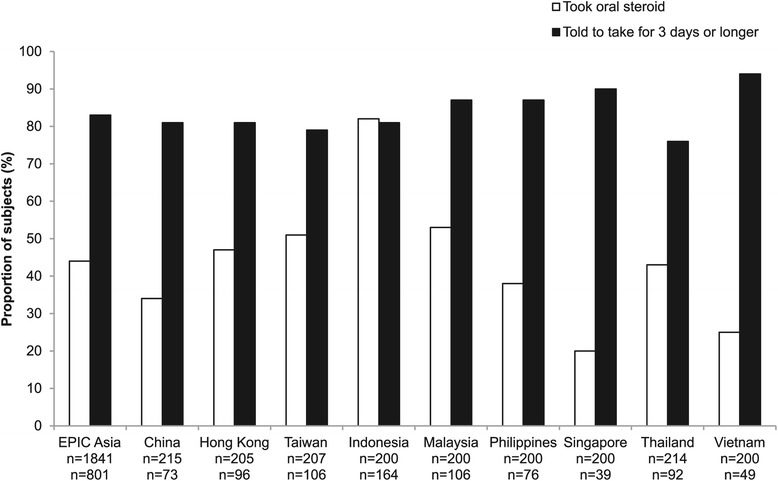
Use of oral corticosteroids. All study subjects (upper row of n values) were asked if they had been told to use oral steroids to manage their respiratory symptoms in the past 12 months. Those who had been prescribed steroids (lower row of n values) were asked if they had been told to take the steroids for three days or longer.

Most subjects reported seeing either a specialist (44%) or general practitioner (34%) for their condition. However, only 37% of study subjects reported that they had been given a lung function test. Of those tested, 89% did not know their test results (either forced expiratory volume in the first second [FEV_1_] value, or percent predicted FEV_1_). Of those who reported taking prescription drugs, 20% did not know the name of the drugs they were taking. When subjects were asked to describe the delivery format of their medication, 57% stated pill/capsule, 13% stated inhaler with spacer, and 12% stated inhaler without spacer (Figure 5). In addition, 44% of the subjects reported the use of oral corticosteroids to manage their symptoms during the previous 12 months (Figure 6). Overall, 35% of the subjects had taken antibiotics for respiratory infections during the previous 12 months, and 13% had received an influenza vaccination.

### Perceptions of disease and attitudes toward physician advice

Subjects were asked questions related to their perception of their condition. More than one-third (35%) felt that there were no truly effective treatments for COPD (Additional file 2: Figure S4A). The majority of subjects (76%) believed that smoking was the cause of their condition, and 70% felt that their condition worsened with increasing age, regardless of treatment (Additional file 2: Figure S4B). However, most subjects felt that with appropriate treatments, progressive increase in breathlessness could be slowed (86%) or they could lead a full and active life (84%) (Additional file 2: Figure S4C).

When asked about the extent to which their doctor’s advice regarding treatment and management helped improve their condition, 43% said ‘a lot’ (Additional file 2: Figure S5A). When subjects were asked about the extent to which their doctor’s advice regarding lifestyle and habits helped, a similar percentage of subjects (39%) said ‘a lot’ (Additional file 2: Figure S5B).

## Discussion

The EPIC Asia survey was a multi-country, cross-sectional, population-based study that examined the prevalence and burden of COPD in the participating Asian territories, from the perspective of individuals who were diagnosed with the disease or who reported symptoms of the disease. This approach contrasts with previous studies in Asia, which have tended to focus on individual countries or areas within countries [15-19].

Using data obtained from the nine Asian territories in this population-based survey, the average prevalence of COPD was estimated at 6.2%. Almost one-fifth of the identified subjects were categorized as having severe or very severe COPD, based on recalled disease classification by a physician, or on their reported symptoms of chronic bronchitis and frequent exacerbations. The prevalence of COPD estimated in this study is similar to that reported by Tan et al. who used a mathematical model to estimate the prevalence of COPD in this region (6.3%) [20]. Similarly, it is consistent with the pooled global prevalence (7.6%) obtained from a meta-analysis of 37 population-based COPD studies using different definitions of COPD including spirometric criteria, patient-reported diagnoses, physician diagnoses, etc. [24]. However, a population-based study which employed spirometric measurements to estimate the prevalence of COPD in 12 countries (the BOLD study), revealed higher COPD estimates (worldwide 10.1%; participating Asian countries 11.4–13.9%) compared with our study. The BOLD study also showed higher levels of severe COPD in the participating Asian countries (1.7–5.0%) [25]. Given that identification of COPD in our study was based on subject-reported physician diagnoses and subjects’ perception of their condition and symptoms, it is likely that the actual prevalence of COPD in the participating Asian territories is higher than was estimated.

The results of this survey may not be directly comparable to those of other COPD studies, due to differences in measurement methodology or study population. Nevertheless, our findings and those of other studies in Asia and elsewhere [15,16,22,24-26] indicate that COPD represents a substantial socioeconomic burden in this region and worldwide. In this study, a large proportion of subjects reported that their condition restricted their work or activities (42%), or kept them from working altogether (23%). Another noteworthy finding is the high rate of hospitalization reported; almost a fifth (19%) of subjects said they had been hospitalized as a result of their condition in the previous year. Unplanned healthcare utilization was also common, with a substantial proportion of subjects visiting a hospital emergency room (26%), or making unscheduled doctor or clinic visits (32%) in the year prior to the survey. In the BOLD study, patients with COPD reported poorer health status than those without COPD; the degree of impairment was greater with increasing COPD severity. Furthermore, patients regarded severe COPD to have a greater negative impact on their health status than diabetes and cardiovascular [26].

Other findings from our study suggest an urgent need for improved clinical management in this region, as well as for better patient education. For instance, a relatively low proportion of subjects had undergone lung function tests (37%), and the majority of those tested did not know their test results (either FEV_1_ value or percent predicted FEV_1_). A fifth of those who reported taking prescription drugs did not know the name of the drugs they were taking. From the perspective of recommended clinical practice, oral corticosteroids appear to be over-prescribed, whereas the use of inhalers is low.

Most studies on COPD are conducted on a selected population who are smokers. Although smoking history is important for COPD diagnosis, environmental pollutants, such as industrial toxins and smoke from biomass fuels, are also highly relevant risk factors, particularly in the Asia-Pacific region [5-7]. Further, smoking status tends to be under-reported [27]. Inclusion of both self-reported smokers and non-smokers in this survey allows the capture of important information on subjects who are smokers but do not accurately report their smoking status, or those whose COPD may be caused by environmental pollutants.

Our findings need to be interpreted within the limitations of the study. Firstly, identification of COPD in subjects was based on subject-reported physician diagnoses, where available, or on the presence of self-reported respiratory symptoms. Similarly, classification of disease severity was based either on subjects’ recall of GOLD classification by a physician, where available, or on their recall of symptomatic criteria. As this was a community survey, subjects were screened based on their reported information; there was no confirmation of diagnoses via subject diaries or spirometric measurements. Consequently, there is potential underdiagnosis, as well as misclassification of COPD and disease severity due to recall bias and subjects’ misperception of their disease condition or symptoms. This may lead to less reliable prevalence estimates. Secondly, subjects who were interviewed via the telephone were likely to have higher social economic status than those who had face-to-face interview. This could result in a selection bias. Potential bias was minimized by conducting random sampling both in regions with high coverage of fixed telephone lines, as well as in those areas without telephone access.

Another limitation of this study is the low response rate which could have introduced the potential for response bias. Subjects were unable to participate in the survey due to various reasons which included ineligible or refusal to participate, contact failure, and inability to complete survey. Hence, it was not possible to collect demographics information from the non-respondents to assess if response bias exists. Nonetheless, steps were taken to mitigate potential bias. Households were randomly selected by RDD or area probability sampling. In households where more than 1 subject was eligible, random sampling was performed to select only 1 subject. In addition, multiple contact attempts were made to reduce contact failure. Another limitation of this study is that the sample size of each country may not be large enough to allow meaningful conclusions to be drawn about each country. Nevertheless, the study enrolled a near uniform number of subjects in each country that minimized the likelihood that the overall prevalence would be affected by unbalanced sample size in any country.

## Conclusions

The results of the EPIC Asia population-based survey suggest a high prevalence of COPD in the participating Asia-Pacific territories, and indicate a substantial socioeconomic burden of the disease in this region. Individuals with the disease reported substantial limitations in their daily activities and loss in work productivity. These findings highlight the need to enhance patient and physician education, and improve the management of COPD in this region.

## Additional files

Additional file 1: Table S1. Sampling frame for the EPIC Asia survey, by country. Subjects from each of the nine participating territories were sampled either by telephone, using random digit dialing (RDD), or face-to-face (FF) interviews in their local language, to identify individuals who had either received a physician diagnosis of COPD or who met the symptomatic criteria used (see Methods). Additional file 2: Figure S1. Frequency of COPD symptoms experienced over the previous 12 months. Proportion of subjects who experienced the following COPD symptoms at least twice per week in their worst month over this period: (A) being awakened at night either by coughing or shortness of breath, (B) coughing or shortness of breath during the day, (C) coughing up phlegm or wheezing, or (D) tightness of the chest or coughing, wheezing, shortness of breath, or chest tightness due to physical exertion. **Figure S2.** Exacerbation symptoms. The types of symptoms reported to be elevated during exacerbation episodes, as reported by subjects who experienced exacerbations in the 12 months prior to the survey. **Figure S3.** Restriction of daily activities by COPD symptoms. The proportion of subjects who reported that their symptoms impose ‘some’ limitation or limit their activities ‘a lot’ with respect to: (A) sports and recreation and normal activities such as walking, (B) social activities or sleeping, and (C) housekeeping chores or their sex life. **Figure S4.** Subjects’ attitudes to COPD. The proportion of subjects who ‘agreed’ or ‘agreed strongly’ that: (A) there are no truly effective treatments for their condition; (B) smoking is the cause of their condition, and that their condition tends to get worse as they get older; (C) with proper treatment, a progressive increase in breathlessness can be slowed, and that most people with the condition can live a full and active life. **Figure S5.** Subjects’ attitudes to their physician’s advice. The proportion of subjects who reported that their physician’s advice on (A) management and treatment, or (B) modification of lifestyle and habits, improved their ability to manage their respiratory symptoms to the following degrees: ‘Not at all’, ‘Only a little’, ‘Some’, or ‘A lot’. Results are shown only for subjects who provided valid answers to the questions.
