# Supplementary material for: Impact of chronic obstructive pulmonary disease (COPD) in the Asia-Pacific region: the EPIC Asia population-based survey
Source: Asia Pac Fam Med. 2015 Apr 23;14(1):4. doi: 10.1186/s12930-015-0020-9 (PMC4416253; doi:10.1186/s12930-015-0020-9)
Supplement: Additional file 1: Table S1. — Sampling frame for the EPIC Asia survey, by country. Subjects from each of the nine participating territories were sampled either by telephone, using random digit dialing (RDD), or face-to-face (FF) interviews in their local language, to identify individuals who had either received a physician diagnosis of COPD or who met the symptomatic criteria used (see Methods). [file 12930_2015_20_MOESM1_ESM.pdf]

Table S1 – Sampling frame

Territory		Interview method	Language	Interview length (minutes)	Households screened	Individuals aged 40+ screened	Final sample size
North Asia	China	RDD	Mandarin	31	17,498	8,674	215
	Hong Kong	RDD	Cantonese, English	29	15,520	6,291	205
	Taiwan	RDD	Taiwanese	29	16,134	6,631	207
Southeast Asia	Indonesia	FF	Bahasa Indonesia	51	9,158	6,053	200
	Malaysia	RDD and FF	Malay, Mandarin	34	22,140	16,367	200
	Philippines	FF	Tagalog	51	13,925	10,308	200
	Singapore	FF	English	39	8,163	7,368	200
	Thailand	FF	Thai	53	6,563	5,512	214
	Vietnam	FF	Vietnamese	48	3,229	2,375	200
				Mean: 41	Total: 112,330	Total: 69,279	Total: 1,841

RDD = random digit dialing; FF = face-to-face.

Additional file 1

Table S1 - Sampling frame for the EPIC Asia survey, by country

Subjects from each of the nine participating territories were sampled either by telephone, using random digit dialing (RDD), or face-to-face (FF) interviews in their local language, to identify individuals who had either received a physician diagnosis of COPD or who met the symptomatic criteria used (see Methods).
