# Supplementary material for: Impact of chronic obstructive pulmonary disease (COPD) in the Asia-Pacific region: the EPIC Asia population-based survey
Source: Asia Pac Fam Med. 2015 Apr 23;14(1):4. doi: 10.1186/s12930-015-0020-9 (PMC4416253; doi:10.1186/s12930-015-0020-9)
Supplement: Additional file 2: Figure S1. — Frequency of COPD symptoms experienced over the previous 12 months. Proportion of subjects who experienced the following COPD symptoms at least twice per week in their worst month over this period: (A) being awakened at night either by coughing or shortness of breath, (B) coughing or shortness of breath during the day, (C) coughing up phlegm or wheezing, or (D) tightness of the chest or coughing, wheezing, shortness of breath, or chest tightness due to physical exertion. Figure S2. Exacerbation symptoms. The types of symptoms reported to be elevated during exacerbation episodes, as reported by subjects who experienced exacerbations in the 12 months prior to the survey. Figure S3. Restriction of daily activities by COPD symptoms. The proportion of subjects who reported that their symptoms impose ‘some’ limitation or limit their activities ‘a lot’ with respect to: (A) sports and recreation and normal activities such as walking, (B) social activities or sleeping, and (C) housekeeping chores or their sex life. Figure S4. Subjects’ attitudes to COPD. The proportion of subjects who ‘agreed’ or ‘agreed strongly’ that: (A) there are no truly effective treatments for their condition; (B) smoking is the cause of their condition, and that their condition tends to get worse as they get older; (C) with proper treatment, a progressive increase in breathlessness can be slowed, and that most people with the condition can live a full and active life. Figure S5. Subjects’ attitudes to their physician’s advice. The proportion of subjects who reported that their physician’s advice on (A) management and treatment, or (B) modification of lifestyle and habits, improved their ability to manage their respiratory symptoms to the following degrees: ‘Not at all’, ‘Only a little’, ‘Some’, or ‘A lot’. Results are shown only for subjects who provided valid answers to the questions. [file 12930_2015_20_MOESM2_ESM.pdf]

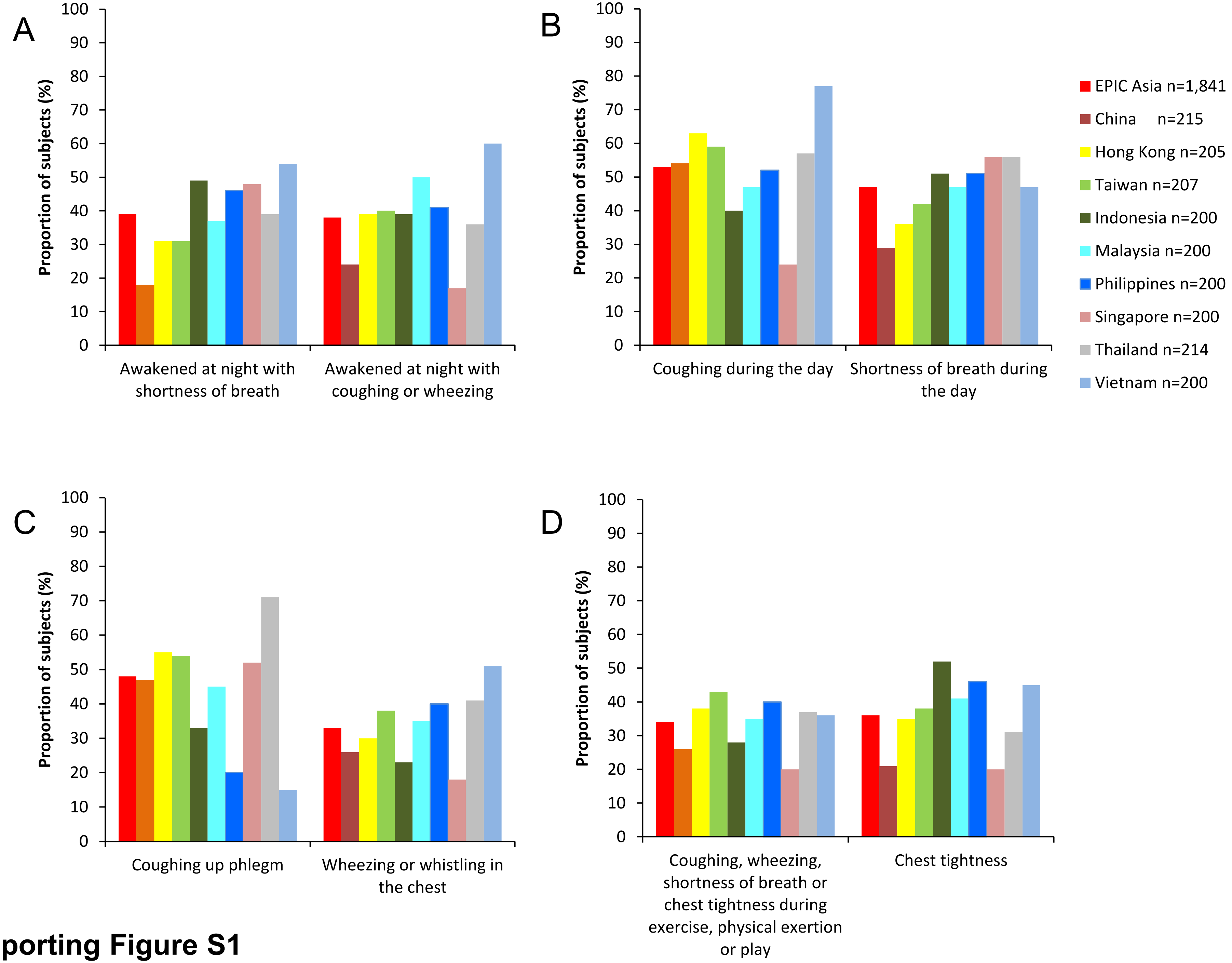

Supporting Figure S1

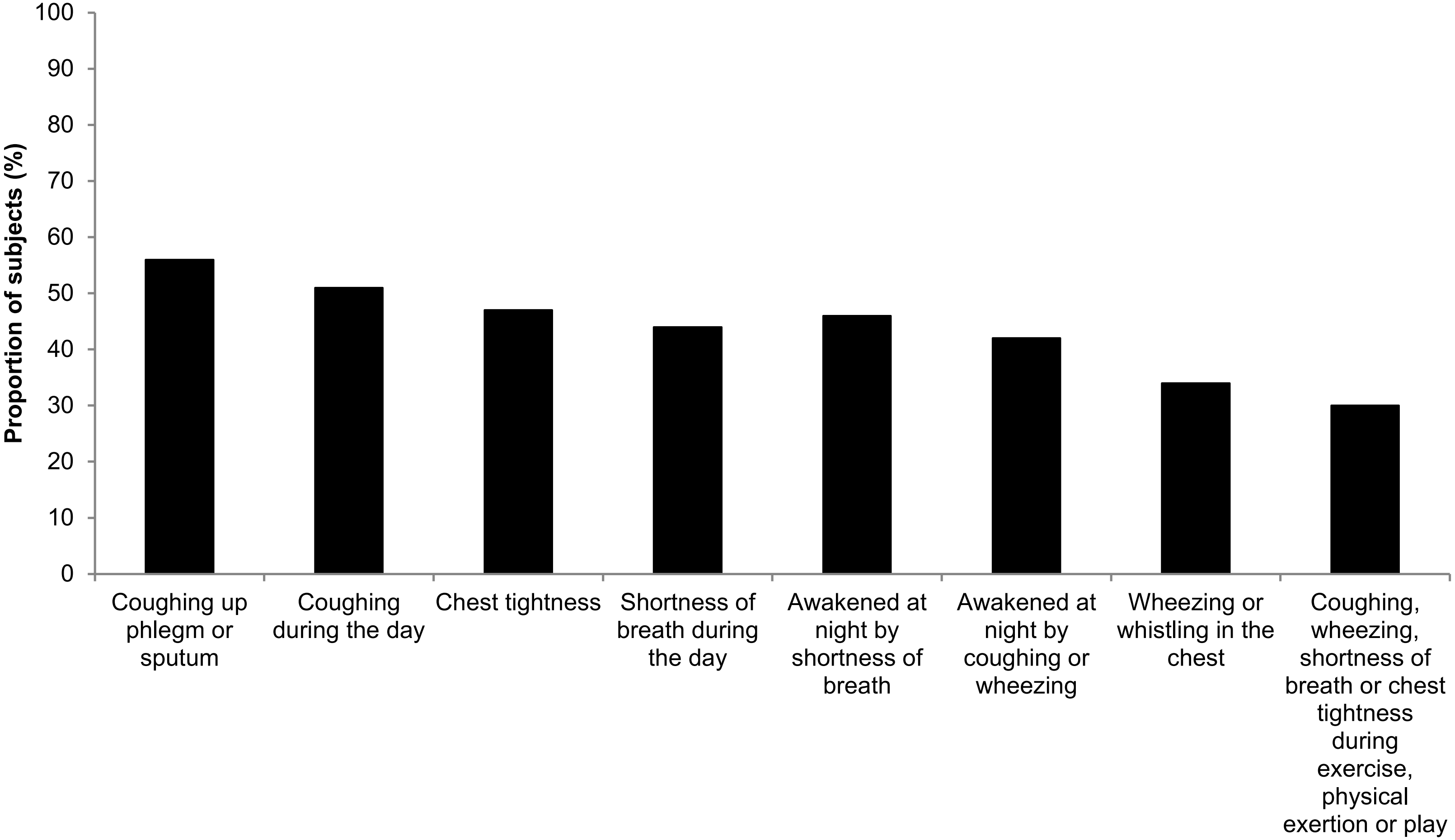

Supporting Figure S2

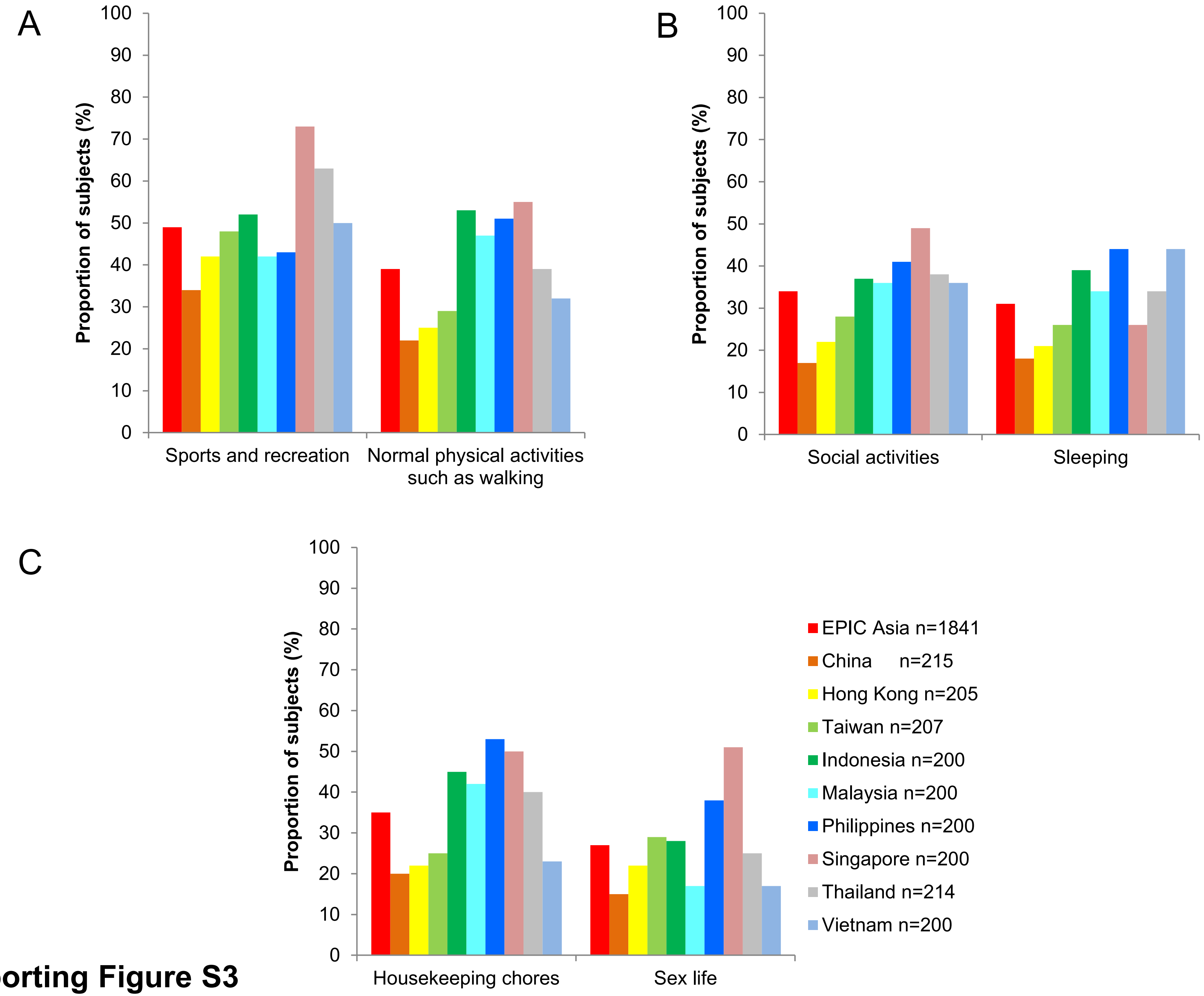

Supporting Figure S3

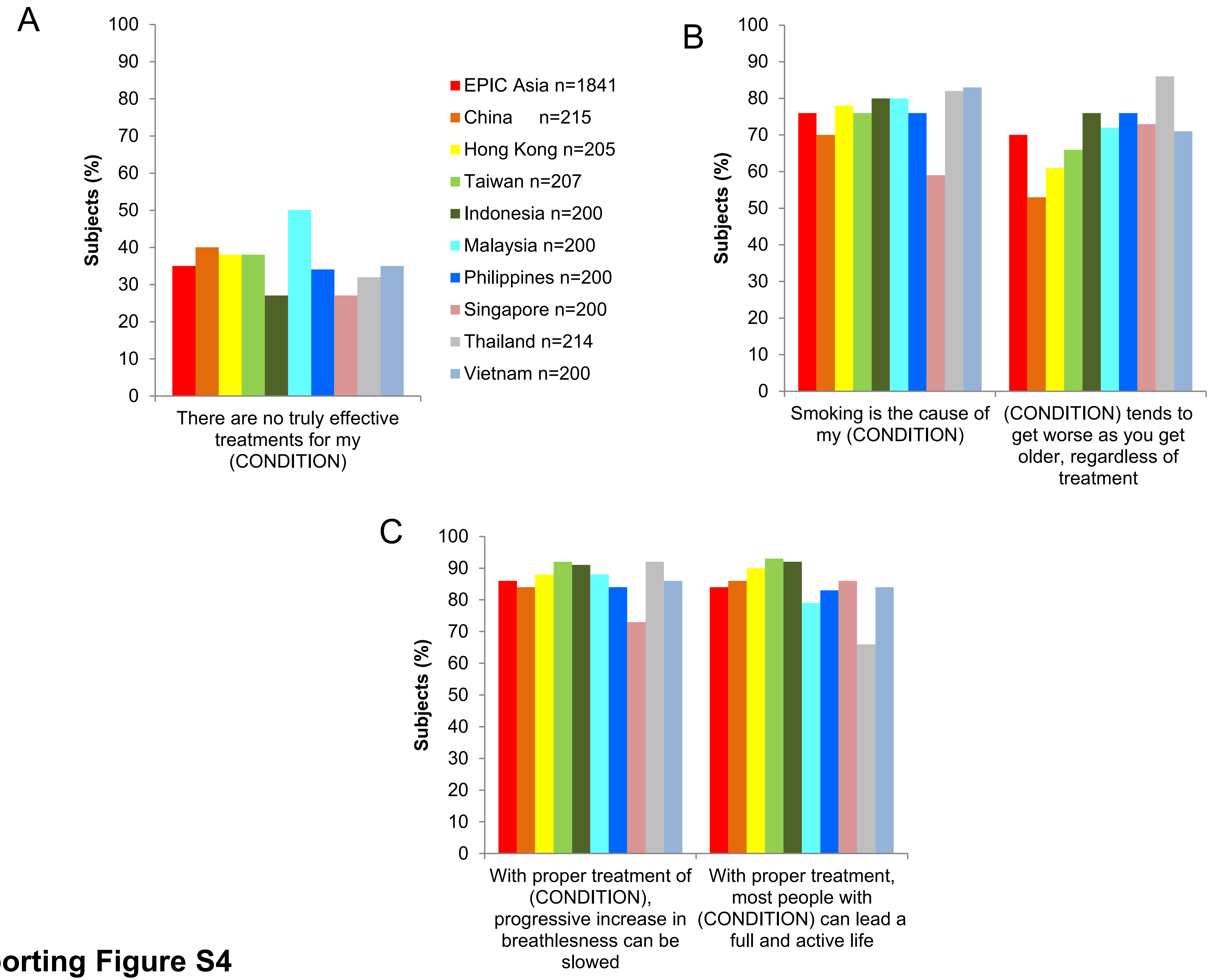

Supporting Figure S4

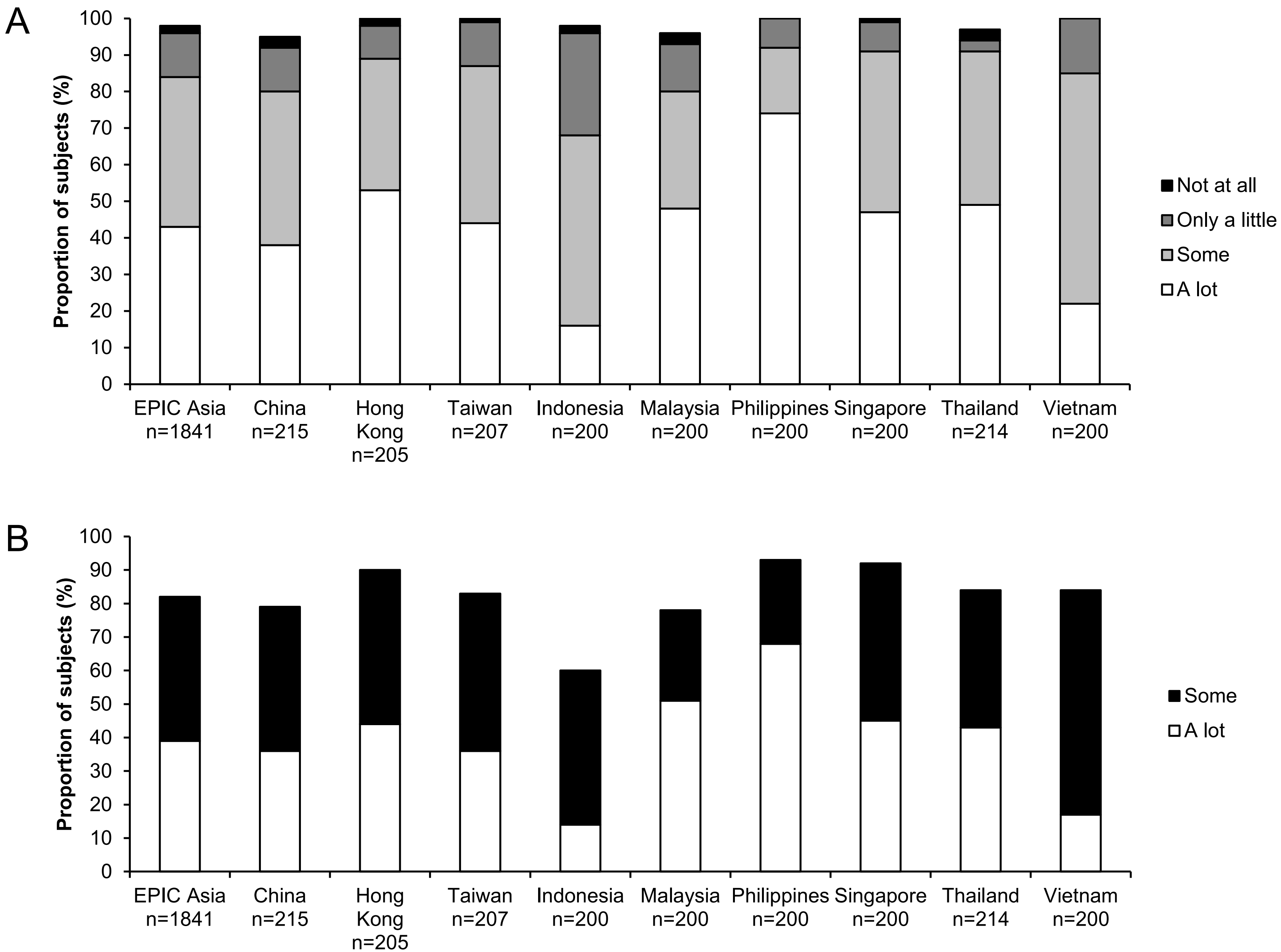

Supporting Figure S5

Additional file 2

Supporting Figure legends

Supporting Figure S1 – Frequency of COPD symptoms experienced over the previous 12 months.

Proportion of subjects who experienced the following COPD symptoms at least twice per week in their worst month over this period: (A) being awakened at night either by coughing or shortness of breath, (B) coughing or shortness of breath during the day, (C) coughing up phlegm or wheezing, or (D) tightness of the chest or coughing, wheezing, shortness of breath, or chest tightness due to physical exertion.

Supporting Figure S2 – Exacerbation symptoms.

The types of symptoms reported to be elevated during exacerbation episodes, as reported by subjects who experienced exacerbations in the 12 months prior to the survey.

Supporting Figure S3 – Restriction of daily activities by COPD symptoms.

The proportion of subjects who reported that their symptoms impose 'some' limitation or limit their activities 'a lot' with respect to: (A) sports and recreation and normal activities such as walking, (B) social activities or sleeping, and (C) housekeeping chores or their sex life.

Supporting Figure S4 – Subjects' attitudes to COPD.

The proportion of subjects who 'agreed' or 'agreed strongly' that: (A) there are no truly effective treatments for their condition; (B) smoking is the cause of their condition, and that their condition tends to get worse as they get older; (C) with proper treatment, a progressive increase in breathlessness can be slowed, and that most people with the condition can live a full and active life.

Supporting Figure S5 – Subjects' attitudes to their physician's advice.
The proportion of subjects who reported that their physician's advice on (A) management and treatment, or (B) modification of lifestyle and habits, improved their ability to manage their respiratory symptoms to the following degrees: 'Not at all', 'Only a little', 'Some', or 'A lot'. Results are shown only for subjects who provided valid answers to the questions.
